# Translational profiling of dorsal root ganglia and spinal cord in a mouse model of neuropathic pain

**DOI:** 10.1016/j.ynpai.2018.04.001

**Published:** 2018-04-18

**Authors:** Sonali Uttam, Calvin Wong, Inês S. Amorim, Seyed Mehdi Jafarnejad, Shannon N. Tansley, Jieyi Yang, Masha Prager-Khoutorsky, Jeffrey S. Mogil, Christos G. Gkogkas, Arkady Khoutorsky

**Affiliations:** a Department of Anesthesia, McGill University, Montreal, QC H3A 0G1, Canada; b Patrick Wild Centre and Centre for Discovery Brain Sciences, University of Edinburgh, Edinburgh EH8 9XD, UK; c Department of Biochemistry and Goodman Cancer Research Centre, McGill University, Montreal, QC H3A 1A3, Canada; d Department of Physiology, McGill University, Montreal QC H3G 1Y6, Canada; e Department of Psychology, McGill University, Montreal QC H3A 1B1, Canada; f Alan Edwards Centre for Research on Pain, McGill University, Montreal QC H3A 0G1, Canada

## Abstract

Acute pain serves as a protective mechanism, guiding the organism away from actual or potential tissue injury. In contrast, chronic pain is a debilitating condition without any obvious physiological function. The transition to, and the maintenance of chronic pain require new gene expression to support biochemical and structural changes within the pain pathway. The regulation of gene expression at the level of mRNA translation has emerged as an important step in the control of protein expression in the cell. Recent studies show that signaling pathways upstream of mRNA translation, such as mTORC1 and ERK, are upregulated in chronic pain conditions, and their inhibition effectively alleviates pain in several animal models. Despite this progress, mRNAs whose translation is altered in chronic pain conditions remain largely unknown. Here, we performed genome-wide translational profiling of dorsal root ganglion (DRG) and spinal cord dorsal horn tissues in a mouse model of neuropathic pain, spared nerve injury (SNI), using the ribosome profiling technique. We identified distinct subsets of mRNAs that are differentially translated in response to nerve injury in both tissues. We discovered key converging upstream regulators and pathways linked to mRNA translational control and neuropathic pain. Our data are crucial for the understanding of mechanisms by which mRNA translation promotes persistent hypersensitivity after nerve injury.

## Introduction

Chronic pain debilitates over twenty percent of the population worldwide, and is the leading cause of long-term disability in humans (Souza et al., 2017). The most common chronic pain conditions include headache, low back pain, cancer pain, arthritis pain, and neuropathic pain, which can result from damage to peripheral nerves or to the central nervous system itself. In addition to dysfunction of the somatosensory system, chronic pain has multi-dimensional effects on the emotional and mental health of patients that can lead to depression, anxiety, sleep disorders, low self-esteem, and impairments in attention and memory (Duenas et al., 2016). Pain management depends largely on antidepressants, anticonvulsants, and opioids; however, pain relief is incomplete under most circumstances and is achieved only in a fraction of patients (Foley, 2003; Kalso et al., 2004; Højsted and Sjøgren, 2007; Moulin et al., 2007; Ballantyne and Shin, 2008).

The inadequate management of chronic pain is a consequence of our incomplete understanding of the mechanisms underlying the induction and maintenance of pain states, leading to treatments that only target symptomatology without addressing the etiology of the disease. Sensitization of nociceptive circuits, both in the central and peripheral nervous systems, leads to mechanical hypersensitivity (allodynia), which is a hallmark of many chronic pain conditions. This sensitization is supported by the expression of new genes, which are required for the biochemical and structural reorganization of the pain pathway. With advancements in microarray and sequencing technologies, transcriptional changes associated with chronic pain have been extensively studied, providing important insights into the transcriptional landscape and identification of a subset of genes with differential expression in various chronic pain conditions (LaCroix-Fralish et al., 2011; Hu et al., 2016; Ray et al., 2017).

Cellular abundance of proteins is highly controlled at the level of mRNA translation (Schwanhausser et al., 2011). Translational control is a powerful modulator of protein levels by regulating the efficiency by which mRNA is converted to proteins.

Translation control involves a variety of mechanisms, including regulation of the vast translational machinery and modulation of the signaling pathways upstream of translation. The extracellular signal-regulated kinase (ERK) pathway and mechanistic target of rapamycin complex 1 (mTORC1) kinase and its downstream effectors have been extensively studied to understand the contribution of translation in the development of hypersensitivity (Khoutorsky and Price, 2017). Suppressing translation by inhibition of mTORC1 reduces mechanical hypersensitivity associated with inflammation (Price et al., 2007; Gregory et al., 2010; Ferrari et al., 2013) and neuropathic pain (Geranton et al., 2009; Zhang et al., 2013). A recent study described an important role for eukaryotic translation initiation factor 2 (eIF2) in inflammation-induced pain, and identified that phosphorylation of the α subunit of eIF2 (eIF2α) is a key step in controlling noxious heat sensitivity (Khoutorsky et al., 2016). Other studies have established a key role for local translation from pre-existing mRNAs in the modification of axonal/dendritic proteomes to promote the excitability of sensory neurons and induce pain hypersensitivity (Melemedjian et al., 2010; Khoutorsky and Price, 2017; Moy et al., 2017b). Altogether, these studies support an emerging role for translational regulation in the establishment and maintenance of chronic pain.

**Fig. 1.**
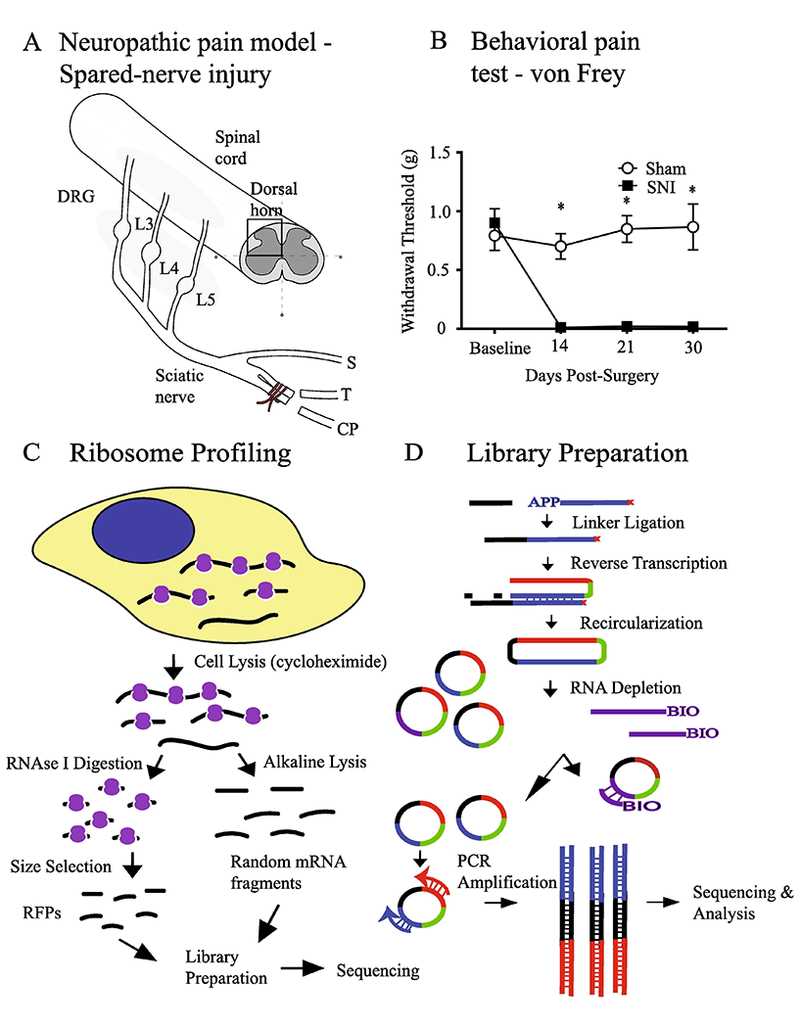
Analysis of gene-expression in the mouse model of neuropathic pain using ribosome profiling and RNA sequencing. (A) A schematic illustration of the SNI assay of neuropathic pain. L3, L4, L5: Lumbar 3,4 and5 level DRG, respectively; S: Sural branch, T: Tibial branch and CP: Common peroneal branch. (B) Paw-withdrawal threshold (g) measured for SNI and sham-operated animals at baseline and 14, 21 and 30 days post-surgery. Symbols represent mean ± SEM; *n* = 8/condition. *p < 0.05 compared to other condition. (C) Experimental flowchart of ribosome profiling technique. (D) Library generation steps of ribosome profiling.

Neuropathic pain accounts for ~20% of chronic pain cases (Lisi et al., 2015), and arises from damage to the nervous system. This damage can result either from a direct injury to peripheral nerves, spinal cord, or the brain, or be caused by a disorder affecting the somatosensory system such as metabolic stress, autoimmunity, degenerative or chronic inflammation, or from idiopathic origin (Guha and Shamji, 2016). Various rodent assays, mostly involving surgical injury, have been developed to study neuropathic pain (Mogil, 2009). Spared nerve injury (SNI) is a model of sympathetic-independent neuropathic pain with long-term chronicity (Decosterd and Woolf, 2000). SNI typically involves a lesion of the tibial and common peroneal branches of the sciatic nerve, while leaving the sural branch intact (Fig. 1A). This procedure causes severe and persistent (at least 6 months) neuropathic pain in the animal, manifested in the sural territory of the ipsilateral paw as mechanical and cold hypersensitivity (Decosterd and Woolf, 2000).

In this study, we have adopted a genome-wide approach to identify mRNAs that are either significantly up- or down-regulated at the level of translation after SNI. For this purpose, we implemented a high throughput RNA sequencing-based methodology, called ribosome profiling, in parallel with measurements of mRNA levels. We analyzed lysates from DRGs and spinal cord (SC) dorsal horn tissues from mice subjected to SNI and mapped the translational and transcriptional landscapes. In addition, we carried out meta-gene analysis by Ingenuity Pathway Analysis (IPA) and identified commonly affected pathways.

## Results

To understand the global pattern of translational control, and identify which mRNAs are differentially regulated following nerve injury, we performed genome-wide translational profiling of DRG and dorsal horn of the spinal cord in the SNI assay of neuropathic pain. For the analysis, we collected L3 to L5 DRG and the corresponding lumbar segment of the spinal cord (Rigaud et al., 2008) 30 days post-SNI. The dorsal half of the spinal cord was dissected and used for the analysis as sensory processing is restricted to this area (illustrated in a schematic diagram in Fig. 1A). We confirmed that mechanical thresholds, as measured by the von Frey test, were significantly reduced at 30 days after the nerve injury (Fig. 1B). Thus, we reasoned that the 30 day time point was appropriate for tissue collection in order to study the chronic phase of neuropathic pain.

To quantitatively measure *in vivo* genome-wide translational efficiency of mRNAs in DRG and spinal cord, we adopted the ribosome profiling methodology (Ingolia et al., 2012). Ribosome/RNA complexes were isolated from cell lysates and digested with an endoribonuclease (RNase I), which degrades all RNAs that are not protected by bound ribosomes (Fig. 1C). This generated ~30 nucleotide long fragments of ribosome-protected mRNAs, or “footprints”. These footprints were reverse-transcribed and cloned into a cDNA library for RNA sequencing (RNA-seq) (Fig. 1D). Libraries were then sequenced to measure the number of footprints per mRNA for the entire genome. Supplementary Table 1 shows the total number of sequenced reads and number of filtered reads (reads uniquely mapped to non-ribosomal region of reference genome DNA) for each sample. In parallel, transcriptome analysis (mRNA-seq) was performed in parallel to account for changes in mRNA abundance. Thus, using the number of footprints (as a proxy for translation) for a given mRNA, normalized to its abundance (as a proxy for transcription), we can calculate translational efficiency (TE) for each mRNA, which has been previously shown to be a strong predictor of protein abundance (Ingolia et al., 2009).

**Fig. 2.**
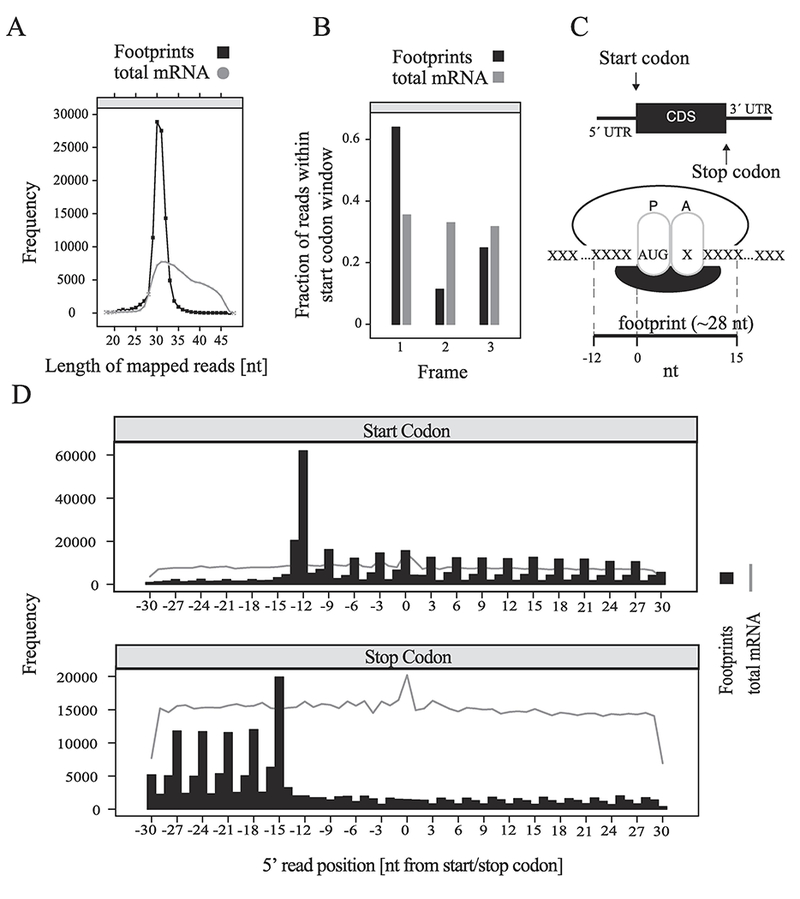
Quality control of ribosome profiling. (A) Frequency of mapped reads from RNA-seq data corresponding to ribosomal footprints (~ 28–32 nt) or total RNA fragments following alkaline fragmentation (~ 28–45 nt). (B) Fraction of reads within start codon window for each one of the three possible frames for footprints and total mRNA. (C) Top: Depiction of a eukaryotic mRNA with 5′ and 3′ UTRs, CDS (coding sequence) and start and stop codons. Bottom: Depiction of the P and A sites on a translating ribosome showing the size and orientation of, and the area occupied by, a typical eukaryotic ribosomal footprint. The start codon AUG is shown; X: any three nucleotides corresponding to a codon. (D) Frequency of footprints and mRNA reads with respect to position from the start (top) and stop (bottom) codons.

Footprints had a narrow size distribution, with a peak corresponding to 28–32 nucleotides, whereas the length of sequencing reads from randomly lysed mRNA fragments as a result of alkaline fragmentation had a broad size distribution ranging from 28 to 45 nucleotides (Fig. 2A) (Ingolia et al., 2009). mRNA-Seq reads were equally distributed between the three possible frames for the start codon, whereas footprint reads displayed a bias for the canonical Frame 1 (Fig. 2B). Likewise, because the size of the protected ribosomal footprint is ~28 nt (Fig. 2C), extending from −12 to +15 (0 being the start codon at the P site of the ribosome), reads around the start codon, stop codon and within the coding sequence follow the periodicity of mRNA codons (3 nucleotides) (Ingolia et al., 2009) (Fig. 2C). As expected, the footprints were largely restricted to the coding sequence (CDS), while the mRNA fragment reads were evenly distributed throughout the 5′ untranslated region (5′ UTR), CDS and the 3 UTR (Fig. 2D). The three-nucleotide periodicity of the ribosome footprints (RFPs) (Fig. 2D), as well as the significantly higher number of RFP reads within the coding region, as compared to UTRs, demonstrates the specificity of the recovered ribosome footprints.

**Fig. 3.**
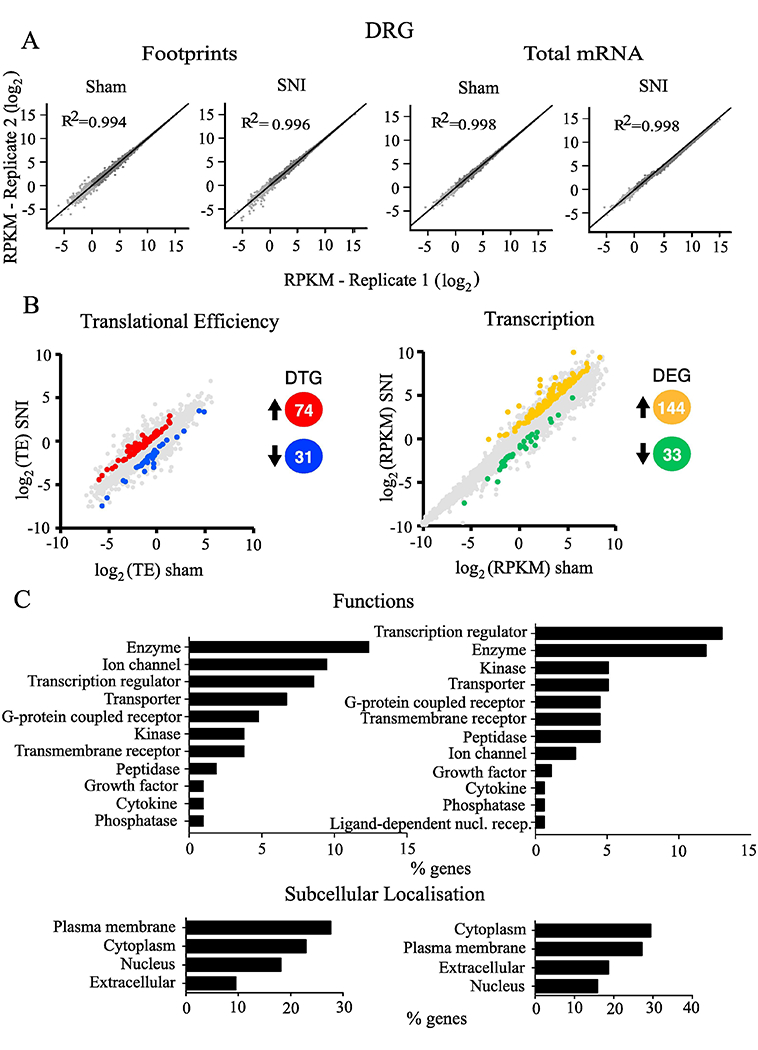
The DRG translational and transcriptional landscape after SNI. (A) Correlation between replicates for footprint (left) and total mRNA (right) are shown for sham or SNI groups in DRG. (B) Changes (log_2_) in translational efficiency (left) and transcription (right) and differentially translated or transcribed genes (upregulated and downregulated; *p* < 0.05 and 0.5 > fold change > 1.5) between sham- and SNI-treated mice are depicted from ribosome profiling analysis in tissue from DRG. The number of differentially translated genes (DTG) or differentially expressed genes (DEG) is depicted in different colours (red/blue, orange/green). Spearman’s rank correlation coefficient (R^2^) is shown for log_2_ comparisons. (C) Representative functional analysis characteristics using IPA of differentially regulated genes at the level of translation (left) and transcription (right) in DRG, 30 days post-SNI. (For interpretation of the references to colour in this figure legend, the reader is referred to the web version of this article.)

**Fig. 4.**
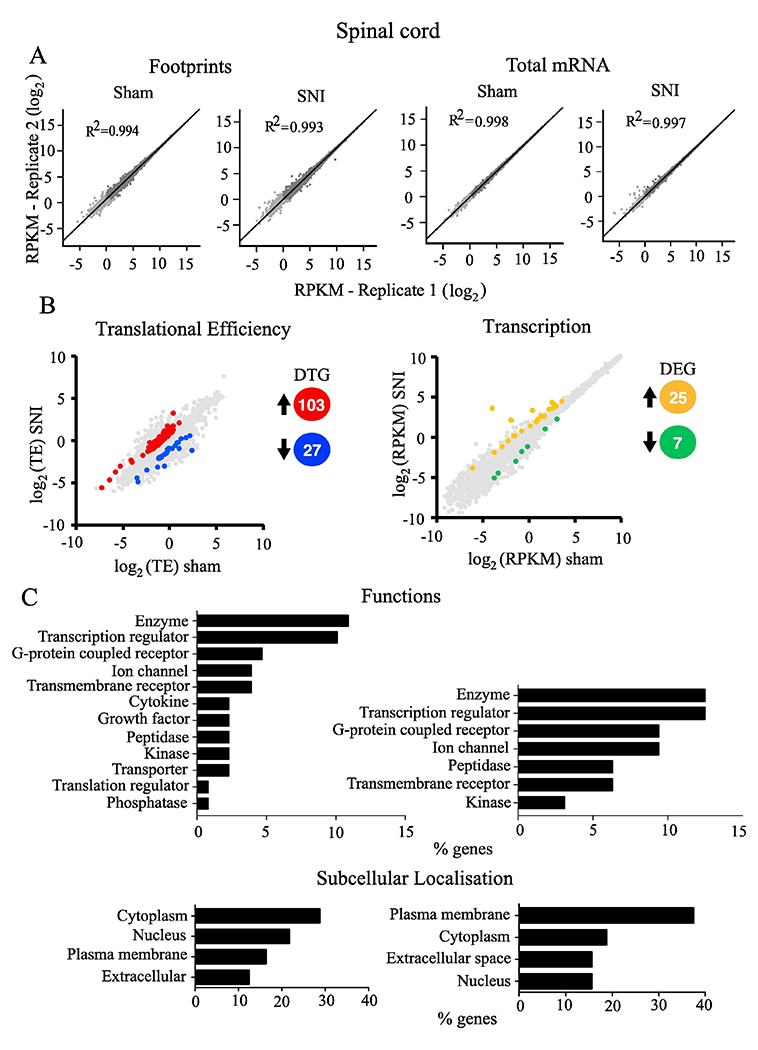
The dorsal horn of the spinal cord translational and transcriptional landscape after SNI. (A) Correlation between replicates for footprint (left) and total mRNA (right) are shown for sham or SNI groups for spinal cord. Spearman’s rank correlation coefficient (R^2^) is shown for log_2_ comparisons. (B) Changes (log_2_) in translational efficiency (left) and transcription (right) and differentially translated or transcribed genes (upregulated and downregulated; *p* < 0.05 and 0.5 > fold change > 1.5) between sham and SNI treated animals are depicted from ribosome profiling analysis in spinal cord. The number of differentially translated genes (DTG) or differentially expressed genes (DEG) is depicted with different colors (red/blue, orange/green). (C) Representative functional analysis characteristics using IPA of differentially regulated genes at the level of translation (left) and transcription (right) are shown for sham or SNI groups in spinal cord, 30 days post-SNI. (For interpretation of the references to colour in this figure legend, the reader is referred to the web version of this article.)

Footprints and mRNA densities were computed in units of reads per kb per million (RPKM) to normalize for gene length and total reads per sequencing run. All conditions demonstrated a strong correlation between biological replicates (Figs. 3A and 4A – R2; Pearson Correlation). Based on changes in translational efficiency, 74 mRNAs were upregulated (fold change > 1.5, *p* < 0.05) in the DRG of SNI mice as compared to sham animals, while translation was downregulated (0.5 > fold change, *p* < 0.05) for 31 mRNAs (Fig. 3B left, for the complete list of genes see Supplementary Table 2). mRNA-seq analysis revealed that 144 mRNA were transcriptionally upregulated and 33 were downregulated in DRG after SNI (Fig. 4B right, for the complete list of genes see Supplementary Table 2). In the spinal cord, 103 mRNAs were translationally upregulated and 27 were downregulated (Fig. 4B left, for the complete list of genes see Supplementary Table 2), whereas 25 mRNAs were transcriptionally upregulated and 7 were downregulated after SNI (Fig. 3B right, for the complete list of genes see Supplementary Table 2).

**Fig. 5.**
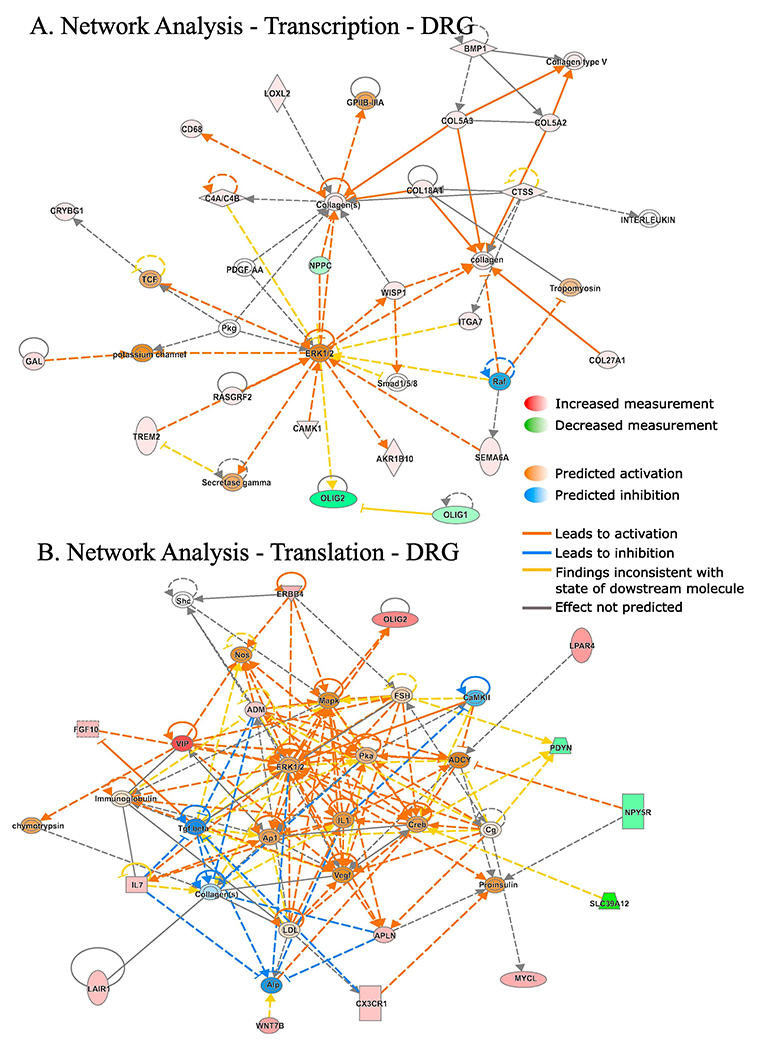
Network analysis generated by IPA of differentially transcribed and translated mRNAs in DRG 30 days post-SNI. Red: increased measurement; green: decreased measurement; orange: predicted activation; blue: predicted inhibition; yellow: findings inconsistent with state of downstream molecule; grey: effect not predicted; solid line: direct interaction; dashed line: indirect interaction. (For interpretation of the references to colour in this figure legend, the reader is referred to the web version of this article.)

Ingenuity Pathway Analysis (IPA) of differentially regulated genes (both translationally and transcriptionally) in SNI revealed top cellular functions and subcellular localizations in the DRG (Fig. 3C) and spinal cord (Fig. 4C). We also used the IPA network analysis of differentially regulated genes to generate a node graph of potential regulatory networks based on the ribosome profiling data for DRG (Fig. 5) and spinal cord (Supplementary Fig. 1).

## Discussion

Translational control of gene expression has emerged as a prominent mechanism in the regulation of gene expression in pathological pain states (Price and Geranton, 2009; Melemedjian and Khoutorsky, 2015; Khoutorsky and Price, 2018). Indeed, signaling upstream to the translation machinery is upregulated in several chronic pain conditions (Price et al., 2007; Jimenez-Diaz et al., 2008; Geranton et al., 2009; Ji et al., 2009; Khoutorsky and Price, 2017). Moreover, an inhibition of mRNA translation has been shown to effectively alleviate pain in several preclinical assays (Geranton et al., 2009; Asante et al., 2010; Obara et al., 2011). Despite this progress, the repertoire of mRNAs showing altered translation in pain conditions remains largely unknown. Our study provides the first genome-wide translational profiling of DRG and spinal cord tissues in a mouse model of neuropathic pain. We identified 74 mRNAs in DRG and 103 mRNAs in the spinal cord whose translation is increased 30 days following SNI, and 31 mRNAs in DRG and 27 mRNAs in the spinal cord with decreased translation. The higher number of upregulated versus downregulated mRNAs in DRG after SNI is consistent with previous studies showing increased signaling upstream of translation following nerve injury in DRG (Obata et al., 2004; Price et al., 2007; Khoutorsky et al., 2016; Moy et al., 2017a) and increased rates of translation in sensory neurons in response to pronociceptive stimulation (Melemedjian et al., 2010). The parallel analysis of changes in mRNA levels and their translational efficiency demonstrates that changes in these processes occur in the opposite direction for multiple mRNAs, suggesting translational buffering (Laurent et al., 2010; McManus et al., 2014) (see Supplementary Table 3). For example, in the DRG, seven genes (*Myh* 7, *Mobp,* 1500009C09Rik, *Sall1, Grin2b, O1ig2* and 3110035E14Rik) are transcriptionally down regulated but translationally upregulated. In the spinal cord, four genes (*Scn4a, Htr3b, Sprr1a* and *Rtn4rl2*) are transcriptionally upregulated but translationally down regulated, whereas *Tmem54* is transcriptionally downregulated but translationally upregulated. Several genes that have been previously studied in relation to pain show opposite changes in mRNA levels and their translation efficiency (spinal cord: *Scn4a, Htr3b, Sprr1a, Rtn4r12, Tmem54;* DRG: *Myh7, Mobp,* 1500009C09Rik, *Sall1, Grin2b, Olig2* and 3110035E14Rik). For example, *Scn4a* gene codes for the alpha subunit of the voltage-dependent sodium channel, and mutations in this gene have been associated with sodium channel myotonia (Orstavik et al., 2015). *Htr3b* codes for the serotonin-3B receptor. *Htr3b* rs1176744 polymorphisms are proposed to influence and predict the development of chronic pain disorders like chronic myalgia (Louca Jounger et al., 2016). In a transcriptomic analysis of human DRG, *Sprr1a* (small proline-rich protein 1a) was identified as a signature gene associated with pain experienced in sickle cell disease (Paul et al., 2017). Additionally, *Sprr1a* is involved in regeneration (Jing et al., 2012) and its protein levels are elevated in DRG following peripheral nerve injury (Starkey et al., 2009).

We predict that genes showing changes in the same direction in their mRNA levels and TE, such as *Pkd2l1, Unc45b, Tmem88b* and *Trhr,* will exhibit robust changes in the corresponding protein levels. Polycystic kidney disease protein 2-like 1 (PKD2L1) is a member of the transient receptor potential superfamily which is known to be involved in a number of sensory functions, ranging from detection of light, force, osmolality, temperature, odour, taste, and pain (Hussein et al., 2015). A study identified *Tmem88b* in DRG to be transcriptionally up-regulated following burn injury (Yin et al., 2016). However, the physiological role of *Tmem88b* in sensory neurons and pain remains poorly defined.

To better understand the biological context of the identified genes, we analyzed our datasets using the IPA platform. IPA analysis has categorized the differentially regulated genes in DRG and spinal cord, post-SNI, into functional and subcellular localization categories, identifying several overlapping functions between transcriptionally and translationally regulated genes (Figs. 3C and 4C), including enzyme, transcription regulator, ion channel, and G protein-coupled receptors. Interestingly, the network analysis identified ERK as a central hub of both transcriptionally and translationally controlled genes, depicted by the large number of edges converging and diverging from the node corresponding to ERK (Fig. 5). This finding is in accordance with previous studies establishing the central role of ERK pathway in the development of hypersensitivity associated with both inflammatory and neuropathic pain (Ji et al., 2002; Zhuang et al., 2005). Indeed, in DRG, several vital transcriptional and growth factors, cytokines, and other signaling molecules (i.e., CREB and MAPK) participate in the network by either activating or inhibiting ERK. In response to noxious stimulation, ERK phosphorylates and activates CREB, thus facilitating transcription of CREB-dependent genes, many of which are implicated in pain (Ji et al., 1999). In addition, activation of ERK promotes mRNA translation via mitogen-activated protein kinase interacting kinase (MNK1/2)-dependent phosphorylation of eukaryotic initiation factor 4E (eIF4E), the cap binding protein, which is critical for ribosome recruitment to the mRNA (Waskiewicz et al., 1997; Moy et al., 2017a). This phosphorylation event promotes the excitability of DRG neurons (Moy et al., 2017a) and leads to the enhanced translation of brain-derived neurotrophic factor mRNA in DRG neurons (Moy et al., 2018) which in turn induce translation and transcription of pain-relevant genes. Together, our network analysis provides further evidence for the involvement of ERK in both transcriptional and translational gene networks, supporting the model of feed-forward loops between transcriptional and translational control mechanisms in which the ERK pathway is serving as a central regulatory mechanism.

Changes in transcriptional and translational regulation in the spinal cord could be underrepresented in our analysis, considering that we extracted tissue from the entire dorsal half of the spinal cord, whereas most of the sensory processing is restricted to the dorsal horn area. Since we analyzed lysates prepared form spinal cord and DRG tissues, we most likely detect changes in both neuronal and non-neuronal cellular populations, including infiltrated immune cells. It is also important to note that this study is based on female mice. Since painprocessing mechanisms might differ between sexes (Sorge et al., 2015), similar studies in males, as well in other species, are ultimately required.

In summary, we performed the first translational profiling study of DRG and spinal cord tissues after nerve injury, and identified mRNAs whose translational efficiency is altered in the SNI animal model of neuropathic pain. The IPA analysis revealed altered cellular pathways, including identification of ERK as a key regulator of both translational and transcriptional networks. This information is instrumental for furthering our understanding of the molecular underpinnings of chronic pain.

## Materials and methods

### Neuropathic pain

All procedures involving mice were carried out in compliance with the Canadian Council on Animal Care guidelines and were pre-approved by the McGill University Animal Care Committee. C57BL/6J female mice, at 8 weeks of age, underwent the bilateral SNI surgical procedure as described previously (Decosterd and Woolf, 2000; Shields et al., 2003) to induce neuropathic pain. Briefly, under 2% isoflurane anesthesia, the lateral surface skin of the thigh was shaved and incised. The biceps femoris muscle was incised to expose the sciatic nerve just below its branching point. The tibial and common peroneal branches were tightly ligated using 7-0 silicone coated silk (Covidien, S-1768K) and a 3–1 mm portion of each of the ligated branches was sectioned and removed distal to the ligation point. Finally, the muscle and the skin incisions were closed using 6-0 Vicryl suture (Ethicon, J489G). During the entire process, great care was taken to leave the sural branch unharmed. The mouse was returned to its home-cage for recovery. Sham animals were used as controls, where the surgical procedure was carried out identically but all three branches of the sciatic nerve were left untouched and unharmed. The animals were sacrificed 30 days postsurgery, and DRG and dorsal horn of the spinal cord samples were extracted. Tissues from 10 animals were pooled per sample and 2 independent replicates were made for each of the four conditions.

### Harvesting of DRG and dorsal horn of spinal cord

To collect DRG and dorsal horn of the spinal cord, animals were sacrificed by brief isoflurane anesthesia followed by decapitation. The animal was secured on a bed of dry ice and the spinal cord was exposed and doused with RNA*later* stabilization solution (Ambion, AM7020). Lumbar DRG (level L3-L5) were excised for all animals. Next, the lumbar region of the spinal cord at which the L3-L5 DRG branch into was excised and placed on a bed of dry ice/metal plate and allowed to freeze after which it was cut along the frontal plane to separate the dorsal horn section. The DRG and dorsal horn were collected in non-stick, RNase free microcentrifuge tubes (Ambion, AMI 2450), immediately snap-frozen in liquid nitrogen, and stored at −80 °C until further processing.

### Ribosomal profiling

#### Tissue homogenizadon and cell lysis

Flash frozen DRG and dorsal horn tissue was lysed in ice-cold cell lysis buffer (1% Polysome buffer (20 mM TrisCl (pH 7.4), 150 mM NaCl, 5 mM MgCl_2_, 1 mM DTT and 100 μg/ml cyclohexamide, 8% glycerol), 1% Triton X-100 and 25 U/ml Turbo DNase I) in a glass homogenizer system. The total lysate was divided into two fractions. A fraction containing at least 150 μg of total RNA was reserved for ribosome footprinting (RFP fraction) and the remaining (at least 100 μg) was processed for mRNA-Seq.

#### Obtaining ribosome footprints (RFPs)

Ribosome footprinting was carried out as previously described (Ingolia et al., 2012) with minor modifications. Briefly the RFP fraction was subjected to RNase I treatment (Ambion, AM2295) at a concentration of 2 U/μg of RNA, at 4 °C for 45 min with end over end mixing and quenched for 5 min by adding 4U SUPERaseIn (Ambion, AM2696) for every 5 U of RNase I. Monosomes were pelleted by ultracentrifugation (Beckman Coulter, Optima MAX-UP)) through a 34% sucrose cushion (in polysome buffer) at 70,000 RPM for 3 h at 4 °C. The resulting RNA pellet was resuspended in 600 μl Tris C1 (pH 7) and RNA was extracted by double acid Phenol and one Chloroform extraction, precipitated by 1 vol Isopropanol and 1/9 vol 3 M NaOAc (pH%.5) and 2 μl Glycoblue (15 mg/mg stock, Invitrogen, AM9515) at −80 °C overnight followed by centrifugation at 20,000*g* at 4 °C for 30 min. Purified RNA was resolved on a 15% polyacrylamide urea gel (Invitrogen, EC6885BOX) and bands corresponding to 28–32 nucleotides, containing the desired ribosome footprints (RFPs), was excised and extracted for RNA using Costar Spin-X column (Sigma, CLS8160).

#### Random RNA fragmentation of cytoplasmic RNA

Poly (A)+ mRNAs were purified from 100 μg of cytoplasmic RNA, using magnetic oligo-dT DynaBeads. The purified RNA was then subjected to alkaline fragmentation by treating it with an equal volume of 2× alkaline fragmentation solution (2 mM EDTA, 10 mM Na_2_CO_3_, 90 mM NaHCO_3_, pH 9.2) for 20 min at 95 °C. The reaction was stopped by addition of the precipitation solution (300 mM NaOAc pH 5.5 and 2 μl GlycoBlue), followed by Isopropanol. Fragmented mRNAs were size-selected on a denaturing 10% polyacrylamide-urea gel and the bands corresponding to 30–50 nucleotides were excised, eluted, and precipitated with Isopropanol.

#### Library preparation for sequencing

Fragmented mRNA and RFPs were subjected to PNK dephosphorylation and 10 pmol of the dephosphorylated RNA fragments were used for ligation to a pre-adenylated and 3′-blocked linker, followed by separation on a 10% polyacrylamide urea gel. Linker ligated bands were excised and extracted for RNA, which was reverse transcribed using ONTI223 adapter (Illumina) and Superscript III reverse transcriptase (Invitrogen) according to the manufacturer’s instruction manual. The resulting cDNA was purified by size selection on a 10% polyacrylamide Tris/Borate/EDTA-urea (TBE-urea) gel. The cDNA was then circularized using CircLigase (Epicentre, CL4111K). Products arising from ribosomal sequences were depleted using biotinylated rDNA complementary oligos (Ingolia et al., 2012) and MyOne Streptavidin C1 dynabeads. The remaining products were amplified by PCR (11 cycles) using indexed primers, size-selected on a 8% polyacrylamide gel and purified. At these intermediate steps, bands in the gels that were very close to the fragment size + adapter were excised and purified. The resulting cDNA library samples were analyzed on an Agilent Bioanalyzer High Sensitivity DNA chip to confirm the size and concentration and then sequenced using the non Strand-Specific, single-read 50 (SR50) on the Illumina HiSeq 2500 Sequencing platform according to the manufacturer’s instructions, with sequencing primer ONTI202 (5CGACAGGTTCAGAGTTCTACAGTCCGACGATC).

#### Bioinformatics analysis of ribosomal footprinting data

Raw sequencing data were de-multiplexed by the sequencing facility (Genome Quebec). Sequences were analyzed using a custom developed bioinformatics pipeline adapted from Ingolia et al. (2012) as described in Silva Amorim et al. (2018). In brief, reads were adapter-trimmed, contaminant sequences (rRNA, tRNA) were removed using bowtie with optimised parameters for ribosome profiling as per Ingolia et al. (2009) and reads were aligned to a reference mouse genome (GRCm38.p5). Since the RNA-seq and ribosome footprint assays were paired for each sample of the four conditions (DRG_SNI; spinal cord_SNI; DRG_Sham and spinal cord_Sham), the RNA-seq data were used to normalize the footprint numbers to derive the Translation efficiency (TE).

Reads Per Kilobase of transcript per Million mapped reads (RPKM) was calculated using an in-house R-script described in Ingolia et al. (2009) for each transcript. TE for each transcript was calculated by dividing RPKM values of the RFP libraries by RPKM values of the total mRNA libraries for each of the two sample condition replicates and then averaged. Z-score, P-values and FDR were calculated for all transcripts as in Silva Amorim et al. (2018). Genes with < 128 reads were discarded. A Supplementary Table 4 includes RPKM abundances for all genes for all experiments. Raw RNA-seq data is available upon request.

### IPA

Pathway Analysis was performed using the Ingenuity Pathway Analysis Software (IPA; Qiagen; version 42012434). Datasets previously filtered to include only differentially expressed and differentially translated genes were submitted to IPA. Location and Type information were obtained from the IPA annotated datasets to determine the % of genes from each dataset belonging to individual subcellular localization and molecular type/function categories. Data was plotted as% of genes in each category, with category “other” not shown. IPA annotated datasets were submitted to Core Analysis with analysis parameters set to include “Direct and indirect interactions” and “Experimentally observed data only”. Network data was obtained for all datasets and a Molecular Activity Predictor (MAP) analysis was applied based on the differentially regulated genes belonging to each individual network.

## Supplementary Material

SUpp Table 4

Supp Fig 1

Supp Table 1

Supp Table 2

Supp Table 3
