# Supplementary material for: Translational profiling of dorsal root ganglia and spinal cord in a mouse model of neuropathic pain
Source: Neurobiol Pain. 2018 Apr 18;4:35–44. doi: 10.1016/j.ynpai.2018.04.001 (PMC6428075; doi:10.1016/j.ynpai.2018.04.001)

Supplementary Figure 1

A. Network Analysis - Transcription - Spinal Cord

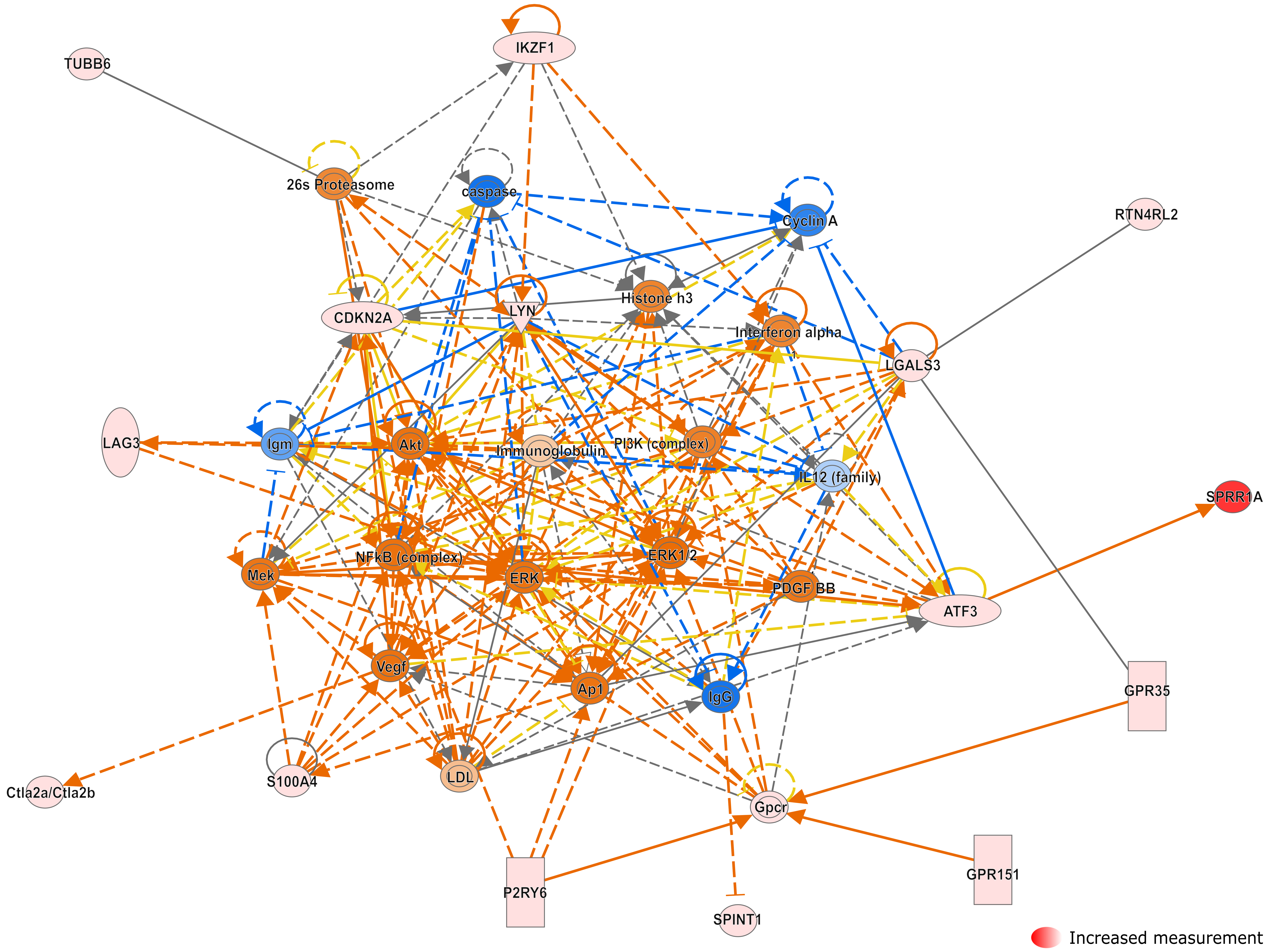

B. Network Analysis - Translation - Spinal Cord

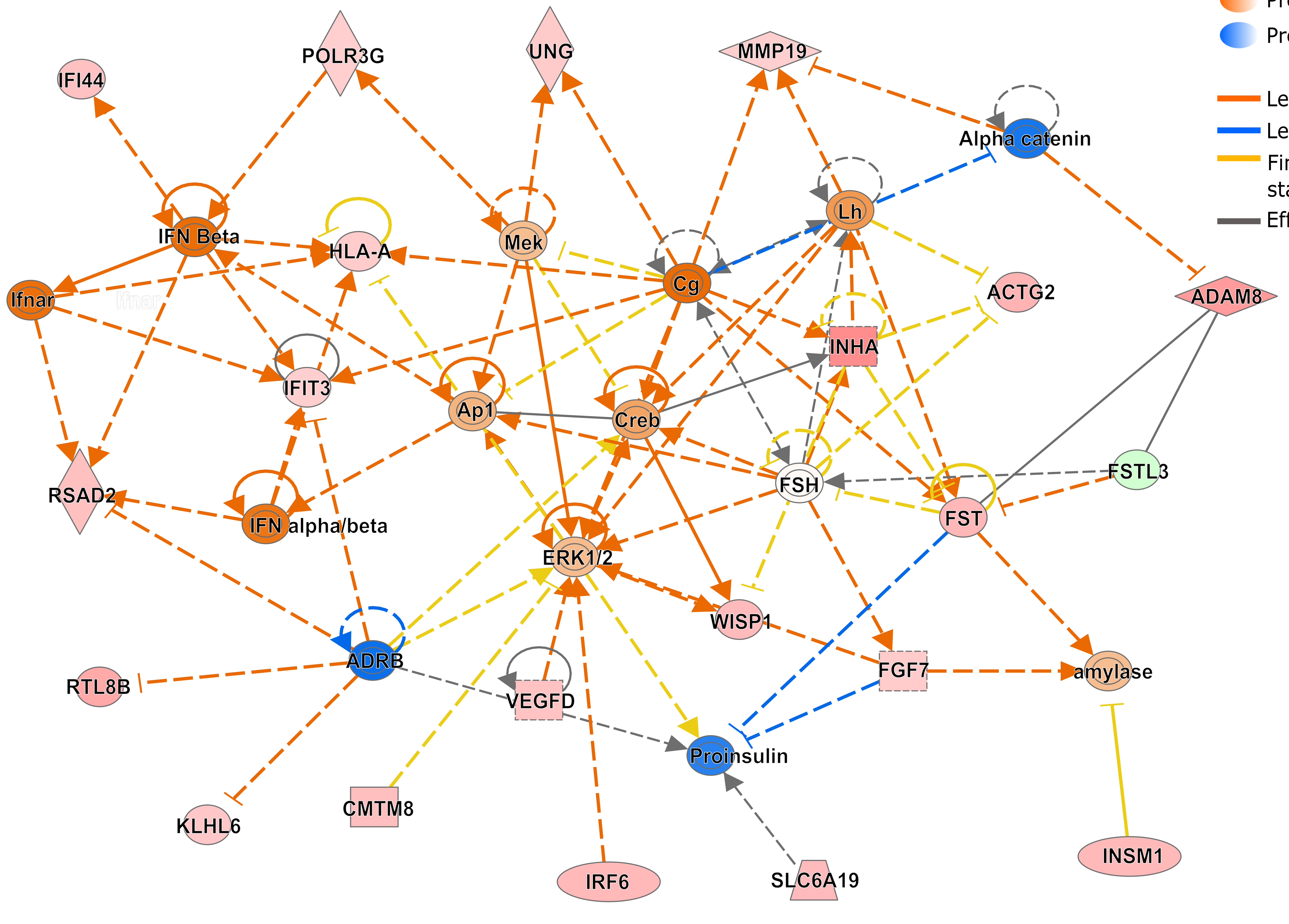

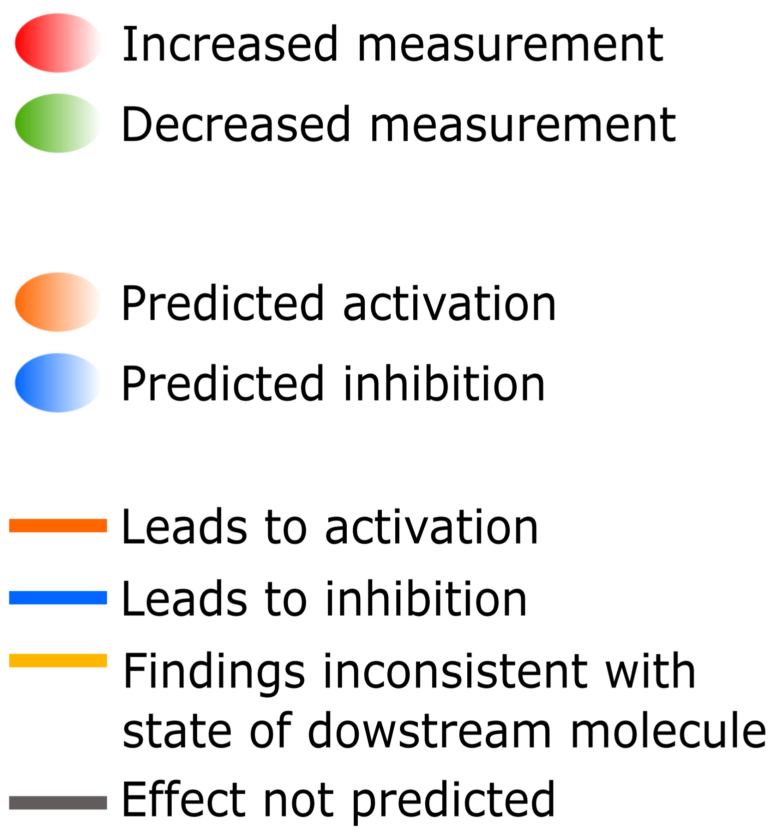

Supplement: Supplementary Fig. 1 — Network analysis of differentially transcribed and translated mRNAs in spinal cord, 30 days post-SNI. Red: increased measurement; green: decreased measurement; orange: predicted activation; blue: predicted inhibition; yellow: findings inconsistent with state of downstream molecule; grey: effect not predicted; solid line: direct interaction; dashed line: indirect interaction. [file mmc6.pdf]
